# Development and interlaboratory validation of a cultivar-specific identification method for the table grape ‘Shine Muscat’ using loop-mediated isothermal amplification (LAMP)

**DOI:** 10.1270/jsbbs.24074

**Published:** 2025-06-21

**Authors:** Reona Takabatake, Yuki Monden, Akiko Shindo, Yasutaka Minegishi, Fumiya Taniguchi, Yu Hashimoto, Tomoyuki Takeuchi, Kazuto Takasaki, Sachiko Isobe

**Affiliations:** 1 Institute of Food Research, National Agriculture and Food Research Organization, 2-1-12 Kannondai, Tsukuba, Ibaraki 305-8642, Japan; 2 Graduate School of Environmental, Life, Natural Science, and Technology, Okayama University, 1-1-1 Tsushimanaka, Kitaku, Okayama, Okayama 700-8530, Japan; 3 Nippon Gene Co., Ltd., 1-5, Kandanishiki-cho, Chiyoda-ku, Tokyo 101-0054, Japan; 4 Institute of Fruit Tree and Tea Science, National Agriculture and Food Research Organization (NARO), Tsukuba, Ibaraki 305-8605, Japan; 5 FASMAC Co., Ltd., 3088 Okata, Atsugi, Kanagawa 243-0021, Japan; 6 Kazusa DNA Research Institute, Kisarazu, Chiba 292-0818, Japan

**Keywords:** Shine Muscat, cultivar identification, LAMP, DNA chromatography, retrotransposon

## Abstract

‘Shine Muscat’ is an elite table grape cultivar developed by the National Agriculture and Food Research Organization in Japan. Recently, the infringement of breeders’ rights in various fruits has become a serious problem in Japan. In this study, a loop-mediated isothermal amplification (LAMP)-mediated cultivar identification method for ‘Shine Muscat’ was developed. We comprehensively analyzed retrotransposon insertion sites using 24 major grape cultivars and identified two insertions, VINE1-Cl160 and VINE1-Cl155, which are unique to ‘Shine Muscat’. LAMP primers targeting VINE1-Cl160 and VINE1-Cl155 were designed, and specific amplifications were confirmed. We also designed a primer set to detect the grape endogenous reference sequence, UDP-glucose:flavonoid 3-*O*-glucosyltransferase. To improve rapidness and cost-effectiveness, we applied single-stranded tag hybridization on a chromatography printed-array strip system, a lateral flow DNA chromatography technology. The developed method was validated with an interlaboratory study. This novel identification method would be particularly useful for border inspections.

## Introduction

‘Shine Muscat’ is a promising table grape cultivar in Japan, bred by the National Agriculture and Food Research Organization (NARO), and known for its large berries with shining yellow green skin, a high sugar content, and edible skin. The cultivation area has been increasing and reaching 2345 hectares in 2021, and it is the second largest among grape cultivars in Japan after ‘Kyoho’ (Ministry of Agriculture, Forestry and Fisheries of Japan (2024).

In general, considerable time and effort are required to develop a new cultivar or to improve breeds, particularly for fruits. ‘Shine Muscat’ took 33 years from the development of its parental cultivar to the registration. Akitsu-21, a parental cultivar of ‘Shine Muscat’, was developed as a hybrid of ‘Steuben’ (*V. labruscana*) × ‘Muscat of Alexandria’ (*V. vinifera*) in 1973. ‘Shine Muscat’ was then selected from a cross of Akitsu-21 and ‘Hakunan’ (*V. vinifera*) in 1988 and finally registered as a new cultivar in 2006 (Yamada *et al.* 2008, 2017).

Recently, ‘Shine Muscat’ seedlings were suspected to have been taken to other countries without permission and cultivated in areas more than 30 times larger than those of Japan (Ministry of Agriculture, Forestry and Fisheries of Japan 2022). In addition, illegal reimportation into Japan from these countries is a major concern. Therefore, a cultivar identification system is necessary to protect breeder’s rights. Currently, to prevent such illegal import, inspections for ‘Shine Muscat’ have been conducted at the border. DNA polymorphic markers, which differ in their DNA sequences among different cultivars, are commonly used for cultivar discrimination or identification, therefore, a rapid and simple DNA marker-mediated identification method is required for ‘Shine Muscat’, which is available even during border inspections.

Retrotransposons are ubiquitous genetic elements that relocate through a “copy and paste” pathway relying on reverse transcription, and the resulting cDNA randomly inserts into a new target site in a genome (Feschotte *et al.* 2002, Kumar and Hirochika 2001, Levin and Moran 2011). Plant genomes have numerous groups of retrotransposons, and their insertions are stably inherited, further, their insertion polymorphisms sometimes show cultivar specificity and can be used as DNA markers usable for cultivar identification (Hirata *et al.* 2020, Monden and Tahara 2017, Naito *et al.* 2014).

DNA chromatography technologies are available for the detection of polymerase chain reaction (PCR) products, such as the Single-stranded Tag Hybridization (STH) for Chromatography Printed-Array Strip (PAS). In the STH C-PAS system, one primer is labeled with a single-stranded specific tag sequence and the other primer is labeled with biotin. After PCR amplification, the C-PAS, a paper chromatograph on which the complementary oligonucleotides of the tag sequences are printed, is dipped into the reaction mixture. The amplification products are immobilized by the single-tag hybridization and visually detected through streptavidin-biotin interactions using streptavidin-coated blue-colored latex microspheres. Several C-PAS-mediated cultivar discrimination and identification methods have been reported (Monden *et al.* 2014, 2023, Okamoto *et al.* 2023).

PCR has been used as the gold standard for various types of genetic testing including cultivar discrimination, however, performing PCR requires a relatively long time along with expensive instruments and reagents. In contrast, loop-mediated isothermal amplification (LAMP) is a rapid, highly specific, and isothermal DNA amplification technique that uses a DNA polymerase with high strand displacement activity (Notomi *et al.* 2000).

We have previously developed several LAMP-mediated genetically modified organism (GMO) detection methods using C-PAS (Takabatake *et al.* 2018a, 2018b, 2023).

In this study, we describe the development of a novel cultivar identification method for Shine Muscat using LAMP and C-PAS, targeting a specific retrotransposon insertion, and the developed method was validated in an interlaboratory study.

## Materials and Methods

### Plant materials

In total, 24 grape cultivars preserved at the Institute of Fruit Tree and Tea Science, NARO consisting of ‘Aki Queen’, ‘Campbell Early’, ‘Kyoho’, ‘Queen Nina’, ‘Grosz Krone’, ‘Koshu’, ‘Concord’, ‘Sunverde’, ‘Shine Muscat’, ‘Suiho’, ‘Steuben’, ‘Sekirei’, ‘Takao’, ‘Delaware’, ‘Niagara’, ‘Nagano Purple’, ‘Pione’, ‘Fujiminori’, ‘Black Beet’, ‘Portland’, ‘Muscat of Alexandria’, ‘Muscat Bailey A’, ‘Ruby Roman’, ‘Rosario Bianco’ were used, these major cultivars account for almost 95% of the grape cultivation area in Japan (e-Stat 2021). Commercial ‘Shine Muscat’ samples were purchased at local markets.

### DNA extraction

Genomic DNA was extracted from grape leaves using an ISOSPIN Plant DNA kit (NIPPON GENE, Tokyo) according to the manufacturer’s instruction. The concentration and quality of the extracted DNA were evaluated by the measuring ultraviolet (UV) absorbance using a NanoDrop One spectrophotometer (NanoDrop Technologies, Wilmington, DE). The concentration of genomic DNA was adjusted to 20 ng/μL, and 20 ng was used as a template for LAMP analyses.

For direct LAMP detection, we used the GenCheck^®^ DNA Extraction Reagent (FASMAC, Kanagawa, Japan). Two hundred μL of lysis buffer was added to 10 mg of grape berry skins, which were heated for 10 min at 100°C, and then chilled on ice. The samples were centrifuged at 15,000 × g for 5 min, and the resulting supernatants were directly used as templates for LAMP analyses.

### Library construction and sequencing

Retrotransposon insertion sites were comprehensively identified using previously described methods (Hirata *et al.* 2020, Monden *et al.* 2023, Okamoto *et al.* 2023). In this study, three retrotransposon families (*VINE1*, *Gret1*, and *Tvv1*) were targeted for sequencing and a sequencing library was constructed for each retrotransposon family (Kobayashi *et al.* 2004, Labra *et al.* 2004, Pelsy and Merdinoglu 2002, Pereira *et al.* 2005, Verriès *et al.* 2000). Genomic DNA was randomly fragmented using NEBNext dsDNA Fragmentase (New England Biolabs, Inc., MA, USA), and forked adaptors were ligated to the fragmented DNA. These forked adaptors were prepared by annealing two different oligos (Forked_Type1 and Forked_Com; Supplemental Table 1). Primary PCR amplification was performed using retrotransposon-specific (VINE1-PBS, Gret1-PPT or Tvv1-1st) and adaptor-specific (AP2) primer combinations with adaptor-ligated DNA as the template (Supplemental Table 1). Nested PCR amplification was performed using tailed PCR primers (P5 and P7) and the primary PCR products as templates. The tailed PCR primers contained the P5 or P7 sequence (Illumina) for hybridization on the sequencing flow cell, the Rd1SP or Rd2SP sequence (Illumina) for sequencing primer-binding sites, and several barcodes for multiplex sequencing. Thus, retrotransposon-specific primers (i.e., D501–D506) comprised a P5 sequence, barcode sequence, Rd1SP sequence, and retrotransposon end sequence, whereas adapter-specific primers (D701–D712) comprised a P7 sequence, barcode sequence, Rd2SP sequence, and adapter sequence. The primer combinations used for each sample are listed in Supplemental Table 2. The PCR products were size-selected (500–1,000 bp) on agarose gels and purified using the QIAquick Gel Extraction Kit (QIAGEN). The purified products were then quantified using the Qubit fluorometer (Invitrogen, Carlsbad, CA, USA), and the size selection range was confirmed using the Agilent 2100 Bioanalyzer (Agilent, Santa Clara, CA, USA). A HiSeq sequencing library was prepared by pooling equal amounts of purified barcoded products from each cultivar. HiSeq reads for analyzing retrotransposon insertion sites were deposited to DDBJ under the accession numbers DRA019223 (VINE1), DRA019224 (Gret1), and DRA019225 (Tvv1).

### Data analysis

The resulting paired-end reads (150 bp) were analyzed following procedures described in previous studies (Hirata *et al.* 2020, Monden *et al.* 2023, Okamoto *et al.* 2023). The obtained reads were analyzed using Maser, a pipeline execution system in the Cell Innovation Program of the National Institute of Genetics (https://cell-innovation.nig.ac.jp/index_en.html). Adaptor trimming and quality filtering (QV ≥30) were performed using Cutadapt (Martin 2011) and FLEXBAR (Dodt *et al.* 2012), respectively. The filtered reads were trimmed to a specific length (50 bp) to cover most of the sequences (Monden *et al.* 2023). Reads with 10 or more identical sequences were reduced to a single sequence in the FASTA format and then clustered using the BLAT self-alignment program (Kent 2002) under the following parameter settings: “-tileSize” = 8, “-minMatch” = 1, “-minScore” = 10, “-repMatch” = –1, and “-oneOff” = 2. This clustering analysis yielded several clusters, each corresponding to a specific retrotransposon insertion site. Next, an optimal threshold was set to evaluate the presence or absence of retrotransposon insertions, if the number of reads in a given cluster at a specific insertion site accounted for <0.1% of all reads for that cultivar, retrotransposons were considered absent from that site. This provided genotyping information for the presence (1) versus absence (0) of retrotransposon insertions in all cultivars. Finally, cultivar-specific insertion sites were screened based on this genotyping information.

### Development of DNA markers for identifying ‘Shine Muscat’

Primers designed to anneal with the retrotransposon sequence (VINE1-PBS primer) and insertion site sequences were used to amplify each insertion site (Supplemental Table 3). These primers were expected to amplify DNA fragments from cultivars harboring retrotransposon insertions. VINE1-PBS and Cl160 primer pairs were used to amplify the Cl160 insertion site (Supplemental Table 3). Similarly, the Cl155 insertion site was amplified using a primer combination of VINE1-PBS and Cl155 (Supplemental Table 3). Each PCR was run in a 10 μL reaction volume containing 0.05 μL of TAKARA ExTaq, 0.2 mM of dNTPs, 1 μL of 10x Ex Taq buffer, 0.2 μM of each primer, and 1 μL of genomic DNA (5 ng/μL). The cycling conditions were as follows: 94°C for 4 min, 30 cycles of 94°C for 30 s, 58°C for 30 s, and 72°C for 30 s, then 72°C for 15 min. PCR products were visualized using 2% (w/v) agarose gel electrophoresis.

### LAMP assay

We used two markers, VINE1-Cl160 (Cl160) and VINE1-Cl155 (Cl155), both of which are retrotransposon insertion sites specific to ‘Shine Muscat’. The grape UDP-glucose:flavonoid 3-*O*-glucosyltransferase (UFGT) gene (GenBank accession number, AB047092) was used as the endogenous control (Kobayashi *et al.* 2001). The primer set for each segment was designed using LAMP Designer 1.16 (PREMIER Biosoft, Palo Alto, CA). The primers used in this study are listed in Table 1. All primers were synthesized by Fasmac. LAMP reactions were performed using a Genie II real-time fluorometer (OptiGene Ltd., Horsham, UK) as described previously (Takabatake *et al.* 2018a, 2023). The primer concentrations were 0.2 μM for F3 and B3, 1.6 μM for FIP and BIP, and 0.8 μM for LoopF and LoopB. The LAMP reaction was conducted using the 2x LAMP Master Mix (NIPPON GENE), according to the manufacturer’s protocol, and was performed at 65°C for 30 min, followed by annealing from 98°C to 80°C. The LAMP products were detected using a fluorescent DNA binding dye. A template free control assay was also performed for all primer sets.

### Signal detection via lateral flow DNA chromatography

The LAMP products were detected using lateral flow DNA chromatography. The reagents for STH C-PAS and the chromatography strips were purchased from TBA (Miyagi, Japan). We used the C-PAS4 membrane, on which four complementary tag sequences (A1, A2, A3, and A4), were printed linearly. In this study, the A1, A2, and A4 tag sequences were used for Cl160, Cl155, and UFGT detection, respectively. The tag sequences were added to the 5′ end of the FIP, BIP, and FIP primers for Cl160, Cl155, and UFGT, respectively. The LoopB, FIP, and BIP primers were biotinylated for Cl-160, Cl155, and UFGT, respectively. The STH C-PAS system was used for the duplex detection of two target sequences such as Cl160 and UFGT or Cl155 and UFGT. For duplex LAMP amplification, two primer sets, meaning a total of 11 primers, were mixed. The primer concentrations for the duplex detection of Cl160 and UFGT were as follows: 0.16 μM for F3 and B3, 1.28 μM for FIP and BIP, and 0.64 μM for LoopB for Cl160; and 0.03 μM for F3 and B3, 0.24 μM for FIP and BIP, and 0.12 μM for LoopF and LoopB for UFGT. The primer concentrations for detecting the duplex of Cl155 and UFGT were as follows: 0.16 μM for F3 and B3, 1.28 μM for FIP and BIP, and 0.64 μM for LoopB for Cl155; and 0.04 μM for F3 and B3, 0.32 μM for FIP and BIP, and 0.16 μM for LoopF and LoopB for UFGT. Signal detection by STH C-PAS was performed as previously described (Takabatake *et al.* 2018b, 2023). The LAMP reaction was conducted using a heating block, DTU-1BN (TAITEC, Saitama, Japan), at 65°C for 40 min. After the LAMP reaction, 1 μL of the LAMP products was diluted with the developing solvent and the C-PAS4 membrane was dipped into the mixture. The LAMP products are then immobilized with single tag hybridization and visually detected with a streptavidin-biotin interaction using a streptavidin-coated blue-colored latex microsphere. Visible blue line(s) appeared after 10–15 min.

### Interlaboratory validation

The interlaboratory study was designed according to ISO13495:2013 (2013) which describes principles of selection and criteria of validation for varietal identification methods using specific nucleic acid, and five laboratories in Japan participated. The experimental protocols and test samples were prepared and provided by the Food Research Institute. A blind test was carried out. Blind samples designed using six different grape cultivars, ‘Kyoho’, ‘Muscat of Alexandria’, ‘Grosz Krone’, ‘Shine Muscat’, ‘Sunverde’, ‘Queen Nina’ were sent to the participants. All participants were first requested to prepare crude extractions for direct LAMP detection from grape berry skin samples using GenCheck^®^ DNA Extraction Reagent. The LAMP reaction was performed on six blind samples and a negative control, in which the reaction was performed without a template in each laboratory using individual instruments at 65°C for 40 min. The instruments used were DTU-1BN, a unit water bath (TAITEC), Cool Stat 5200 (Anatech, Tokyo, Japan), NTS-4000 (EYELA, Tokyo, Japan), and ThermoMixer C (Eppendorf, Hamburg, Germany). The results were considered acceptable when the control lines derived in UFGT were detected from the samples and the negative control was not detected. All participants were requested to submit their data to the Food Research Institute.

## Results

### Sequencing and data analysis of retrotransposon insertion sites

Three retrotransposon families, *VINE1*, *Gret1*, and *Tvv1* were selected for comprehensive analysis of the insertion sites using a high-throughput sequencing platform. In the *VINE1* library, 94,648,193 reads of 150 bp were obtained (min = 1,441,058, average = 3,943,675, max = 6,160,607 reads per line) (Table 2). After preprocessing, 29,204,663 reads remained (Table 3). These reads were used for clustering analysis using the BLAT self-alignment program (Kent 2002), which produced 403 independent insertion sites in 24 cultivars (Table 3). In the *Gret1* library, 86,034,789 reads (min = 1,410,932, average = 3,584,783, and max = 5,539,450 reads per line) of 150 bp were obtained (Table 2). After preprocessing, 21,313,387 reads remained (Table 3). These reads were used for clustering analysis using the BLAT self-alignment program (Kent 2002), which produced 925 independent insertion sites for *Gret1* in 24 cultivars (Table 3). In the *Tvv1* library, 149,157,571 reads (min = 1,894,495, average = 6,214,899, and max = 11,371,555 reads per line) of 150 bp were obtained (Table 2). After preprocessing, 44,211,634 reads remained (Table 3). Cluster analysis of these reads produced 272 independent insertion sites for *Tvv1* in 24 cultivars (Table 3).

We compared the insertion sites of the 24 cultivars and selected suitable insertion sites to discriminate ‘Shine Muscat’ from other cultivars. Among the insertion sites identified in the aforementioned data analysis, we focused on putative insertion sites that were assumed to be present in the target cultivar. PCR primers were designed based on the sequences of the selected insertion sites of *VINE1* (Cl160 and Cl155), and DNA markers derived from the selected insertion sites were investigated for their ability to distinguish ‘Shine Muscat’ from among the 24 cultivars. The Cl160 and Cl155 insertion sites were amplified in ‘Shine Muscat’ but not in other cultivars (Supplemental Fig. 1). Therefore, ‘Shine Muscat’ could be accurately distinguished from the 24 cultivars using either of these markers.

### Specificity evaluation of the primer sets for each target

We used two DNA markers based on retrotransposon insertion polymorphisms, Cl160 and Cl155, to identify ‘Shine Muscat’. The whole genome sequence of ‘Shine Muscat’ is already available (Shirasawa *et al.* 2022). Cl160 and Cl155 correspond from nucleotide 3,809,675 to 3,810,034 in chromosome 13 and from nucleotide 2,302,623 to 2,303,102 in chromosome 9 respectively, in the ‘Shine Muscat’ reference genome sequence VSMuph2.0 (Ichihara *et al.* 2023).

**Fig. 1.**
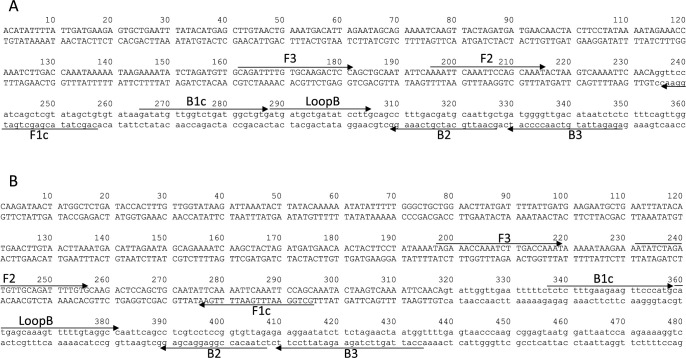
Target sequences of VINE1-Cl160 (A) and VINE1-Cl155 (B). Lowercase letters represent the flanking Shine Muscat genomic sequence and capital letters indicate the insertion site of the retrotransposon sequence. Primers for LAMP are indicated by black arrows. FIP and BIP primers consist of the F2 and B2 primers at the 3ʹ end and F1c and B1c primers at the 5ʹ end, respectively.

**Fig. 2.**
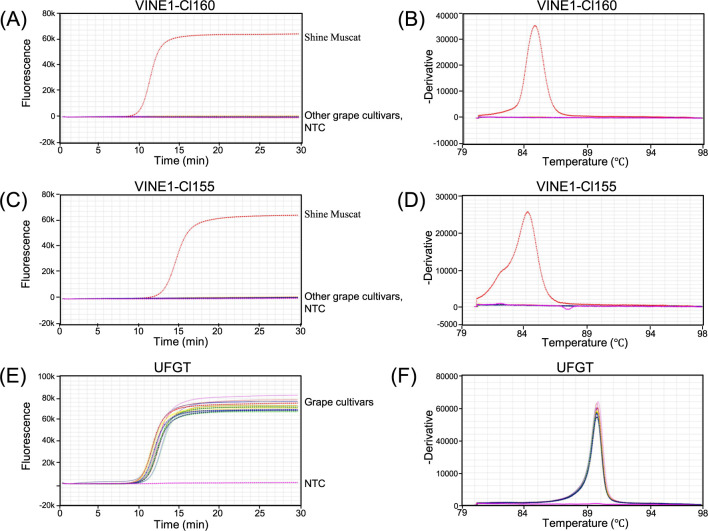
Representative results of the specificity tests for LAMP analyses of the 24 grape cultivars obtained using Genie II. Amplification profiles are shown in (A), (C), and (E), and annealing curves are shown in (B), (D), and (F). The peaks in the annealing curves indicate the annealing temperatures of the LAMP products.

LAMP primers were designed for the junction regions between the retrotransposon insertion sites and flanking genome sequences (Fig. 1). We designed LAMP primers targeting the grape UFGT gene as an internal positive control. First, the specificity of each primer set was evaluated using genomic DNAs extracted from the leaves of 24 grape cultivars. The primer sets used are listed in Table 1. We normally used a set of six primers recognizing eight distinct regions of target sequences comprising F3, B3, F2, B2, F1c, B1c, LoopF, and LoopB for LAMP (Takabatake *et al.* 2018a, 2018b). However, designing eight fully acceptable sequences at the border regions for both Cl160 and Cl155 was difficult. We thus designed F3, B3, FIP, BIP, and LoopB primers for detecting both targets. LAMP products were detected based on the fluorescence intensity using Genie II, a compact, portable instrument. The Genie II system is suitable for real-time fluorescence detection and annealing analyses for amplification products. As shown in Fig. 2A and 2C, amplification was only observed in ‘Shine Muscat’ among the 24 cultivars for both Cl160 and Cl155, indicating that these markers are suitable for ‘Shine Muscat’ identification. Amplifications for UFGT were observed in all cultivars (Fig. 2E).

The annealing curve analyses were performed after LAMP reaction. The annealing temperature was specific to each target sequence, and the analysis was useful for confirming the specificity of the LAMP products. Single peaks were detected for each target, indicating that the primer sets for Cl160, Cl155, and UFGT were specific (Fig. 2B, 2D, 2F).

### Detection using the STH C-PAS system for LAMP products

**Fig. 3.**
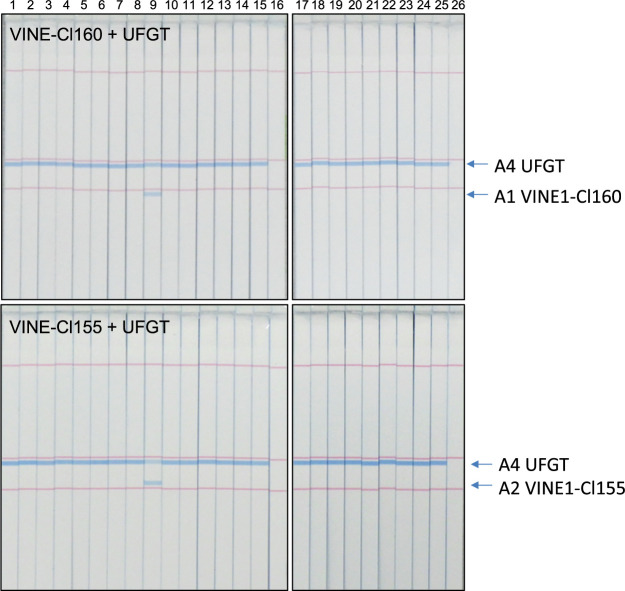
Representative results of specificity tests for LAMP analyses for 24 grape cultivars obtained using STH C-PAS. Lanes 1–15 and 17–25 show the bands for ‘Aki Queen’, ‘Campbell Early’, ‘Kyoho’, ‘Queen Nina’, ‘Grosz Krone’, ‘Koshu’, ‘Concord’, ‘Sunverde’, ‘Shine Muscat’, ‘Suiho’, ‘Steuben’, ‘Sekirei’, ‘Takao’, ‘Delaware’, ‘Niagara’, ‘Nagano Purple’, ‘Pione’, ‘Fujiminori’, ‘Black Beet’, ‘Portland’, ‘Muscat of Alexandria’, ‘Muscat Bailey A’, ‘Ruby Roman’, and ‘Rosario Bianco’, respectively. Lanes 16 and 26, no template control.

To achieve more cost-effective detection, we attempted to develop a multiplex detection using the STH C-PAS system. The C-PAS4 membrane was used, and the A1, A2, and A4 tag sequences were used for Cl160, Cl155, and UFGT detection, respectively. When the two primer sets are mixed and function successfully, different LAMP products can be simultaneously detected. In particular, the duplex detection of a specific marker and a common marker as a positive control is useful. We thus designed duplex detections using combinations of Cl160 and UFGT or Cl155 and UFGT. As the detection sensitivities of each primer set differed, normalization of the two primer sets was required. Finally, we found that the ratios of 0.8:0.15 concentrations for the primer sets of Cl160 and UFGT and 0.8:0.2 concentrations for the primer sets of Cl155 and UFGT, compared to the single LAMP reaction used in Genie II, were the most effective. LAMP reaction was performed using the extracted DNAs from the leaves. As shown in Fig. 3, two clear bands were detected in ‘Shine Muscat’, whereas single bands were detected in the other 23 cultivars.

### Sample direct LAMP detection from grape berry skin

In actual inspections, leaves and fruits are used as samples in many cases. We thus used the grape berries of 10 cultivars, ‘Shine Muscat’, ‘Queen Nina’, ‘Sunverde’, ‘Pione’, ‘Delaware’, ‘Grosz Krone’, ‘Koshu’, ‘Kyoho’, ‘Muscat of Alexandria’, ‘Rosario Bianco’. Direct LAMP detection using crude cell lysates was attempted to simplify and shorten the sample preparation process without requiring DNA extraction and purification steps. In previous studies, the GenCheck DNA Extraction Reagent (FASMAC) was reported to enable sample preparation for LAMP analyses in less than 20 min in a few steps. First, a small amount of the grape berry skin was removed and ground in a microtube. The tubes were heated, chilled on ice, centrifuged, and the resulting supernatants were used as templates. When crude extracts from the 10 cultivars were examined for duplex detection of Cl160 and UFGT or Cl155 and UFGT, two clear bands from both targets were detected only in ‘Shine Muscat’ (Supplemental Fig. 2).

‘Shine Muscat’ was developed by the National Agriculture and Food Research Organization, and we used and analyzed grape leaves and berries derived from the original tree. To evaluate the stability of the two DNA markers, Cl160 and Cl155, we performed an experiment using commercially available ‘Shine Muscat’ samples in markets from different three locations, Fukushima, Nagano, and Okayama prefectures. As shown in Supplemental Fig. 3, amplification signals were detected in all commercial ‘Shine Muscat’ samples from both Cl160 and Cl155. We also checked the sequences of Cl160 and Cl155 and confirmed that both of the sequences were conserved in them (data not shown). These results indicates that the marker sequences are conserved in several different lines of ‘Shine Muscat’.

### Interlaboratory validation of the cultivar identification method

**Fig. 4.**
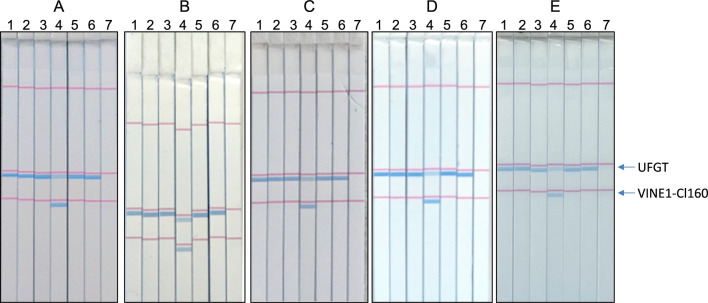
Results of the interlaboratory study in five laboratories. STH C-PAS results of direct LAMP using crude cell lysates are shown. Lanes 1–6 show ‘Kyoho’, ‘Muscat of Alexandria’, ‘Grosz Krone’, ‘Shine Muscat’, ‘Sunverde’, ‘Queen Nina’, respectively. Lane 7, no template control.

To confirm the validity of the developed ‘Shine Muscat’ identification method, an interlaboratory collaborative study was conducted. As one of the criteria for the validation of various identification methods, ISO13495:2013 (2013) proposes an interlaboratory testing with at least four participating laboratories for national level validation using five varieties. We thus conducted an interlaboratory study including five laboratories, A, B, C, D, and E, and provided grape berries of six different cultivars, ‘Kyoho’, ‘Muscat of Alexandria’, ‘Grosz Krone’, ‘Shine Muscat’, ‘Sunverde’, and ‘Queen Nina’ to the participants as blind samples. Cl160 and Cl155 were both highly specific for ‘Shine Muscat’ among the 24 cultivars, but it seemed that the applicability of instruments for Cl160 was better than that for Cl155 (data not shown). We thus used Cl160 for the interlaboratory study. Sample preparation from grape berries using GenCheck DNA Extraction Reagent, LAMP assay, and C-PAS-mediated detection were conducted in each laboratory. The obtained results were shown in Fig. 4 and summarized in Table 4. All participants used simple incubators, such as heating blocks or water baths, for the LAMP reaction. Similar results were obtained from all participating laboratories, indicating that the developed cultivar identification method was reproducible.

## Discussion

In this study, we developed LAMP-mediated cultivar identification method for ‘Shine Muscat’ targeting specific retrotransposon insertion sites. We selected the three retrotransposon families (*Gret1*, *VINE1* and *Tvv1*) for marker development. *Gret1* is a gypsy-like LTR retrotransposon family that was isolated as a retrotransposon-induced mutation in *VvmybA1*, a gene that affects grape skin color (Kobayashi *et al.* 2004). Previous study has shown that *Gret1* is distributed throughout the grapevine genome and that *Gret1* insertion sites are cultivar-specific based on retroelement-microsatellite-amplified-polymorphism (REMAP) and inter-retroelement-amplified-polymorphism (IRAP) banding profiles, suggesting that *Gret1* can be used to develop molecular markers to distinguish cultivars (Pereira *et al.* 2005). *VINE1* is a copia-like LTR retrotransposon that was found to be inserted into the fourth exon of the *Adhr* gene of in various grapevine cultivars (Verriès *et al.* 2000). *VINE1* also exists in multiple copies in the genome and shows polymorphism among cultivars, it is expected that it can be used as an effective molecular tool to distinguish cultivars (Labra *et al.* 2004, Verriès *et al.* 2000). *Tvv1* is a copia-like retrotransposon whose consensus sequence was reconstituted by chromosome walking (Pelsy and Merdinoglu 2002). Previous study demonstrated that internal polymorphism of the UTL region of *Tvv1* is a valuable tool to describe genetic diversity of *Vitis* at the genera, subspecies, species and varieties levels (Pelsy 2007). We investigated comprehensive insertion sites using NGS technology and compared those insertion site among cultivars. Comparison analysis revealed that 71, 46 and 62 cultivar-specific insertion sites were identified in *Gret1*, *Tvv1*, and *VINE1*, respectively, in the 24 grapevine cultivars. Focusing on ‘Shine Muscat’, two, one and four cultivar-specific insertion sites were identified in *Gret1*, *Tvv1* and *VINE1*, respectively. We considered that the discovery of the highest number of ‘Shine Muscat’-specific insertion sites in *VINE1* is a coincidence, and ‘Shine Muscat’-specific insertion sites have also been found in other two families.

There are concerns that the seedlings of newly registered and highly valued cultivars developed in Japan are illegally taken overseas and reimported. As a current method, a technology has been reported to identify 24 grape cultivars containing ‘Shine Muscat’ using simple sequence repeats (SSRs) markers (Yamamoto *et al.* 2019). By the conventional method, 24 grape cultivars including ‘Shine Muscat’ can be identified using 12 markers. However, there are several disadvantages of this method because it is required a fragment analysis using a capillary DNA sequencer and it takes about two days to analyze a few samples. In this study, we comprehensively analyzed the retrotransposon insertion sites in 24 grape cultivar genomes using high-throughput sequencing and identified two specific insertions, Cl160 and Cl155, in ‘Shine Muscat’. These insertions are useful as specific retrotransposon-based markers for the identification of ‘Shine Muscat’ among major domestic cultivars. We further developed a simple, rapid, and cost-effective LAMP-mediated cultivar identification method targeting Cl160 and Cl155. The developed identification method targets markers specific to ‘Shine Muscat’ and is unable to identify grape varieties other than ‘Shine Muscat’. However, ‘Shine Muscat’ is the only grape variety for which an import injunction against importation into Japan has been accepted (Japan Customs 2021). When a suspicious imported fresh grape is found at customs, a rapid identification method which can be determined whether the grapes are ‘Shine Muscat’ or not will be required. By the developed method, the entire detection process, including sample preparation and LAMP detection, was completed within one and a half hours. The current SSR-based method and the developed LAMP-mediated identification method can be individually used for different purposes.

The method developed for Cl160 was validated in an interlaboratory study. The proposed cultivar identification method is expected to be utilized for on-site inspections, and to protect Japanese breeder’s rights regarding Shine Muscat from illegal infringements.

## Author Contribution Statement

RT and Y. Monden contributed equally to this study. RT, Y. Monden, Y. Minegishi, and FT conceived and conceptualized the study. YH and AS provided technical assistance. TT, KT, and SI provided critical advice. RT and Y. Monden wrote the manuscript. The manuscript was reviewed based on the contributions of all the authors. All authors approved the final version of the manuscript.

## Supplementary Material

Supplemental Figures

Supplemental Tables

## Figures and Tables

**Table 1.** Oligonucleotide primers used for LAMP analyses

Target		Sequence
VINE1-Cl160	F3	5ʹ-CAGATTTTGTGCAAGACTCC-3ʹ
	B3	5ʹ-AGAGATTATGTCAACCCCAT-3ʹ
	FIP	5ʹ-CAGCTATACGAGCTGATGGAACAAATTCAAATTCCAGCAAAT-3ʹ
	BIP	5ʹ-GATATGTTGGTCTGATGGCTGTGGCAATTGCATCGTCAAAGG-3ʹ
	LoopB	5ʹ-ATGATGCTGATATCCTTG-3ʹ
VINE1-Cl155	F3	5ʹ-AGAAACCAAATCTTGACCAAAT-3ʹ
	B3	5ʹ-CCATTAGTTCTAGAAGATATTCCTT-3ʹ
	FIP	5ʹ-GCTGGAATTTGAATTTTGAAATATCTAGATGTTGCAGATTTTGTG-3ʹ
	BIP	5ʹ-TCTCTCTTTGAAGAAGTTCCCATGCTAACACCGGAGGACGAG-3ʹ
	LoopB	5ʹ-CATGAGCAAAGTTTTTGTAGGCC-3ʹ
UFGT	F3	5ʹ-TGTTCCCAACACAACCGG-3ʹ
	B3	5ʹ-CCCTCTGGTCTTCTCCAAGA-3ʹ
	FIP	5ʹ-CTACAAGCTCGGCTGGGGGTAGAAAGAAAACCCACCTCGG-3ʹ
	BIP	5ʹ-CTAGCTGAGGCACTGGAGGCTCTGGCAAATGCACCCTTG-3ʹ
	LoopF	5ʹ-GACGGTGCCAAAGCTAATG-3ʹ
	LoopB	5ʹ-CGGGTACCGTTTATATGGTC-3ʹ

**Table 2.** Number of reads in each cultivar

Cultivar name	VINE1	Gret1	Tvv1
Aki Queen	6,160,607	3,875,017	7,207,379
Campbell Early	5,098,552	3,753,653	6,747,361
Kyoho	4,879,087	3,708,825	6,051,474
Queen Nina	4,203,499	3,185,094	5,124,254
Grosz Krone	5,685,185	2,132,978	7,184,484
Koshu	4,855,077	4,464,518	5,670,723
Concord	3,612,348	3,150,112	6,021,484
Sunverde	4,503,994	4,072,960	7,663,586
Shine Muscat	4,699,603	4,207,124	5,545,859
Suiho	4,560,371	3,772,802	6,830,524
Steuben	3,112,210	2,254,087	8,440,093
Sekirei	4,511,269	4,301,931	5,621,816
Takao	4,543,490	5,157,150	6,795,875
Delaware	3,812,699	4,781,821	5,863,528
Niagara	3,125,354	3,695,896	5,768,240
Nagano Purple	3,644,660	3,888,890	4,530,257
Pione	4,753,325	2,551,471	7,297,611
Fujiminori	4,156,124	5,539,450	6,568,370
Black Beet	4,064,153	5,093,287	6,872,058
Portland	2,568,115	3,385,207	4,997,788
Muscat of Alexandria	2,308,302	1,954,233	11,371,555
Muscat Bailey A	2,236,182	2,025,233	2,648,449
Ruby Roman	1,441,058	1,410,932	1,894,495
Rosario Bianco	2,112,929	3,672,118	6,440,308
Total	94,648,193	86,034,789	149,157,571
Minimum	1,441,058	1,410,932	1,894,495
Average	3,943,675	3,584,783	6,214,899
Maximum	6,160,607	5,539,450	11,371,555

**Table 3.** Summary of reads in the data analysis for each family

Retrotransposon	Analysis	No. of reads	Ratio (%)	No. of clusters
VINE1	Raw data	94,648,193	100.00	
	Adaptor removal and QV (≥30) filtering	94,624,449	99.97	
	Trimming to specific length (50 bp)	86,411,790	91.30	
	QV (≥30) filtering	42,861,048	45.28	
	Outlier filtering	29,204,663	30.86	
	BLAT clustering			403
Gret1	Raw data	86,034,789	100.00	
	Adaptor removal and QV (≥30) filtering	86,014,185	99.98	
	Trimming to specific length (50 bp)	80,945,543	94.08	
	QV (≥30) filtering	31,863,975	37.04	
	Outlier filtering	21,313,387	24.77	
	BLAT clustering			925
Tvv1	Raw data	149,157,571	100.00	
	Adaptor removal and QV (≥30) filtering	149,125,620	99.98	
	Trimming to specific length (50 bp)	138,271,067	92.70	
	QV (≥30) filtering	68,316,307	45.80	
	Outlier filtering	44,211,634	29.64	
	BLAT clustering			272

**Table 4.** Summary of the interlaboratory study

Lab	A	B	C	D	E
Amplification of VINE1-Cl160 from Shine Muscat	+	+	+	+	+
Amplification of VINE1-Cl160 from the other 5 grape cultivars	–	–	–	–	–
Amplification of UFGT from the 6 cultivars	+	+	+	+	+
Amplification of VINE1-Cl160 and UFGT from no template control	–	–	–	–	–
Instruments for the LAMP reaction	TAITEC DTU-1BN	TAITEC Unit Water Bath	Anatech Cool Stat 5200	EYELA NTS-4000	Eppendorf ThermoMixer C
