# Supplementary material for: Development and interlaboratory validation of a cultivar-specific identification method for the table grape ‘Shine Muscat’ using loop-mediated isothermal amplification (LAMP)
Source: Breed Sci. 2025 Jun 21;75(3):200–9. doi: 10.1270/jsbbs.24074 (PMC12457787; doi:10.1270/jsbbs.24074)
Supplement: Supplementary file 1 — Supplemental Figures [file 75_200_s1.pdf]

doi: 10.1270/jsbbs.24074

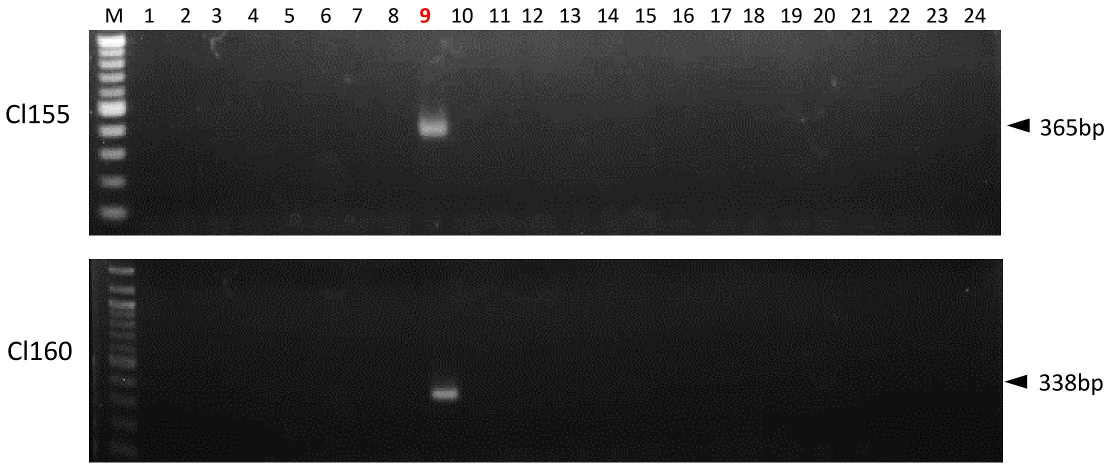

Supplemental Fig. 1. PCR confirmation of cultivar-specific DNA markers. The PCR products were electrophoresed on a 2% agarose gel. The lane numbers correspond to the cultivar names in Table S2. The primer sequences are listed in Table S3.

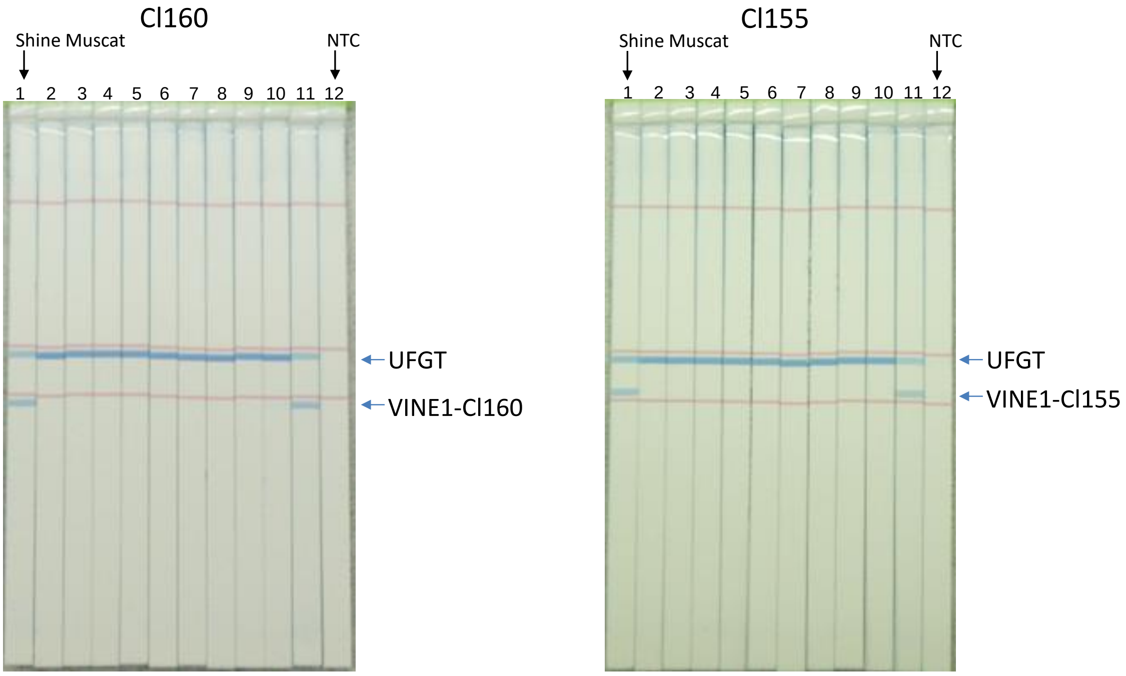

Supplemental Fig. 2. Representative results of sample direct LAMP analyses using crude extracts from the berry skins of 10 grape cultivars. Lanes 1-10, 'Shine Muscat', 'Queen Nina', 'Sunverde', 'Pione', 'Delaware', 'Grosz Krone', 'Koshu', 'Kyoho', 'Muscat of Alexandria', and 'Rosario Bianco', respectively. Lane 11, positive control using genomic DNA extracted from Shine Muscat leaves as the template, and Lane 12, no template control.

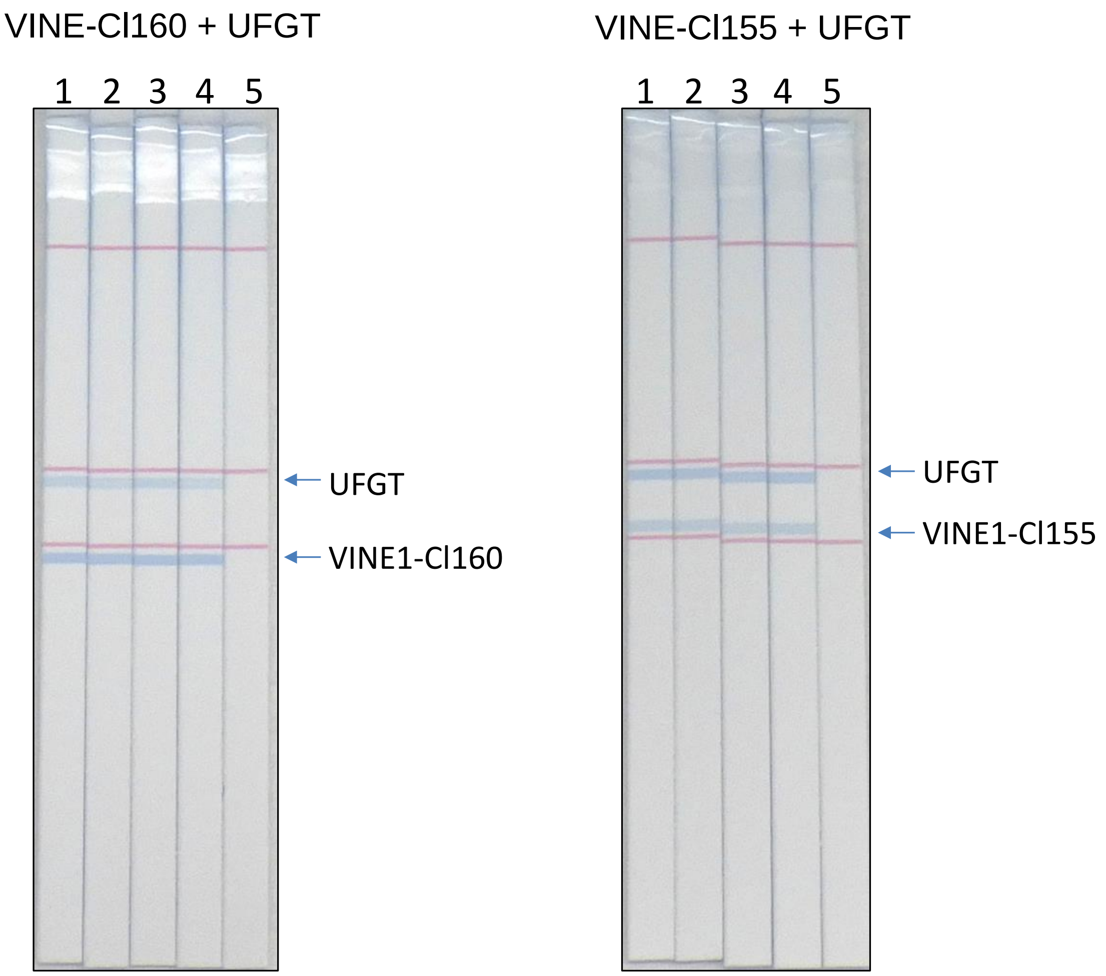

Supplemental Fig. 3. Representative results of sample direct LAMP analyses using 4 Shine Muscat samples including 3 lines purchased in local markets. Lanes 1-4, 'Shine Muscat' derived from original tree, Nagano, Fukushima, and Okayama, respectively. Lanes 5, no template control.
