# Supplementary material for: Development and interlaboratory validation of a cultivar-specific identification method for the table grape ‘Shine Muscat’ using loop-mediated isothermal amplification (LAMP)
Source: Breed Sci. 2025 Jun 21;75(3):200–9. doi: 10.1270/jsbbs.24074 (PMC12457787; doi:10.1270/jsbbs.24074)
Supplement: Supplementary file 2 — Supplemental Tables [file 75_200_s2.pdf]

doi: 10.1270/jsbbs.24074

Supplemental Table 1. Primers used for library construction

Experimental process	Primer name	Sequence (5′→3′)
Adaptor ligation	Forked_Type1[a]	aatagggctcgagcggcagctattaatagtaCt
	Forked_Com[b]	GTACTATTAATAGCATCTTCGTTCGTCGAT
Primary PCR	VINE1-PBS	CAAGATAACTATGGCTCTGATACCA
	Gret1-PPT	AATAGGCAAGTTAAAAAGGGGGGC
	Tvv1-1st	TCCAGCTTGAGGGGGAGTGT
	AP2	AATAGGGCTCGAGCGGC
Nested PCR	D501_VINE1	AATGATACGGCGACCACCGAGATCTACACTATAGCCTACACTCTTTCCCTACACGACGCTCTTCCGATCTCAAATTCCAGCAGATACTAAGTCAAAATTCAACA
	D502_VINE1	AATGATACGGCGACCACCGAGATCTACACATAGAGGCACACTCTTTCCCTACACGACGCTCTTCCGATCTCAAATTCCAGCAGATACTAAGTCAAAATTCAACA
	D503_Gret1	AATGATACGGCGACCACCGAGATCTACACCCTATCCTACACTCTTTCCCTACACGACGCTCTTCCGATCTGCCAAAGACGCGAGGTAACAACA
	D504_Gret1	AATGATACGGCGACCACCGAGATCTACACGGCTCTGAACACTCTTTCCCTACACGACGCTCTTCCGATCTGCCAAAGACGCGAGGTAACAACA
	D505_Tvv1	AATGATACGGCGACCACCGAGATCTACACAGGCGAAGACACTCTTTCCCTACACGACGCTCTTCCGATCTCAAGGAATTGAGAATTACCTTGATTCGGTTACA
	D506_Tvv1	AATGATACGGCGACCACCGAGATCTACACTAATCTTAACACTCTTTCCCTACACGACGCTCTTCCGATCTCAAGGAATTGAGAATTACCTTGATTCGGTTACA
	D701	CAAGCAGAAGACGGCATACGAGATATTACTCGGTGACTGGAGTTCAGACGTGTGCTCTTCCGATCTGGGCTCGAGCGGCAGCTATTAATAGTACT
	D702	CAAGCAGAAGACGGCATACGAGATTCCGGAGAGTGACTGGAGTTCAGACGTGTGCTCTTCCGATCTGGGCTCGAGCGGCAGCTATTAATAGTACT
	D703	CAAGCAGAAGACGGCATACGAGATCGCTCATTGTGACTGGAGTTCAGACGTGTGCTCTTCCGATCTGGGCTCGAGCGGCAGCTATTAATAGTACT
	D704	CAAGCAGAAGACGGCATACGAGATGAGATTCCGTGACTGGAGTTCAGACGTGTGCTCTTCCGATCTGGGCTCGAGCGGCAGCTATTAATAGTACT
	D705	CAAGCAGAAGACGGCATACGAGATATTCAGAAGTGACTGGAGTTCAGACGTGTGCTCTTCCGATCTGGGCTCGAGCGGCAGCTATTAATAGTACT
	D706	CAAGCAGAAGACGGCATACGAGATGAATTCGTGTGACTGGAGTTCAGACGTGTGCTCTTCCGATCTGGGCTCGAGCGGCAGCTATTAATAGTACT
	D707	CAAGCAGAAGACGGCATACGAGATCTGAAGCTGTGACTGGAGTTCAGACGTGTGCTCTTCCGATCTGGGCTCGAGCGGCAGCTATTAATAGTACT
	D708	CAAGCAGAAGACGGCATACGAGATTAATGCGCGTGACTGGAGTTCAGACGTGTGCTCTTCCGATCTGGGCTCGAGCGGCAGCTATTAATAGTACT

D709	CAAGCAGAAGACGGCATACGAGATCGGCTATGGTGACTGGAGTTCAGACGTGTGCTCTTCCGATCTGGGCTCGAGCGGCAGCTATTAATAGTACT
D710	CAAGCAGAAGACGGCATACGAGATTCCGCGAAGTGACTGGAGTTCAGACGTGTGCTCTTCCGATCTGGGCTCGAGCGGCAGCTATTAATAGTACT
D711	CAAGCAGAAGACGGCATACGAGATTCTCGCGCGTGACTGGAGTTCAGACGTGTGCTCTTCCGATCTGGGCTCGAGCGGCAGCTATTAATAGTACT
D712	CAAGCAGAAGACGGCATACGAGATAGCGATAGGTGACTGGAGTTCAGACGTGTGCTCTTCCGATCTGGGCTCGAGCGGCAGCTATTAATAGTACT

Underlined sequences represent barcode sequences.

[a] When a C-T bond was present at the terminus, the oxygen atoms of the phosphate group of the oligonucleotide with normal phosphodiester bonds were exchanged for sulfate atoms to produce phosphorothioate oligonucleotides (S-oligo).

[b] Primers were modified by adding a phosphate group at the 5' terminal.

Supplemental Table 2. Primer combinations used for library construction

Retrotransposon	No.	Cultivar name	P5 primer	P7 primer
VINE1	1	Aki Queen	D501_VINE1	D701
	2	Campbell Early	D501_VINE1	D702
	3	Kyoho	D501_VINE1	D703
	4	Queen Nina	D501_VINE1	D704
	5	Grosz Krone	D501_VINE1	D705
	6	Koshu	D501_VINE1	D706
	7	Concord	D501_VINE1	D707
	8	Sunverde	D501_VINE1	D708
	9	Shine Muscat	D501_VINE1	D709
	10	Suiho	D501_VINE1	D710
	11	Steuben	D501_VINE1	D711
	12	Sekirei	D501_VINE1	D712
	13	Takao	D502_VINE1	D701
	14	Delaware	D502_VINE1	D702
	15	Niagara	D502_VINE1	D703
	16	Nagano Purple	D502_VINE1	D704
	17	Pione	D502_VINE1	D705
	18	Fujiminori	D502_VINE1	D706
	19	Black Beet	D502_VINE1	D707
	20	Portland	D502_VINE1	D708
	21	Muscat of Alexandria	D502_VINE1	D709
	22	Muscat Bailey A	D502_VINE1	D710
	23	Ruby Roman	D502_VINE1	D711
	24	Rosario Bianco	D502_VINE1	D712
Gret1	1	Aki Queen	D503_Gret1	D701
	2	Campbell Early	D503_Gret1	D702
	3	Kyoho	D503_Gret1	D703
	4	Queen Nina	D503_Gret1	D704
	5	Grosz Krone	D503_Gret1	D705
	6	Koshu	D503_Gret1	D706
	7	Concord	D503_Gret1	D707
	8	Sunverde	D503_Gret1	D708
	9	Shine Muscat	D503_Gret1	D709

	10	Suiho	D503_Gret1	D710
	11	Steuben	D503_Gret1	D711
	12	Sekirei	D503_Gret1	D712
	13	Takao	D504_Gret1	D701
	14	Delaware	D504_Gret1	D702
	15	Niagara	D504_Gret1	D703
	16	Nagano Purple	D504_Gret1	D704
	17	Pione	D504_Gret1	D705
	18	Fujiminori	D504_Gret1	D706
	19	Black Beet	D504_Gret1	D707
	20	Portland	D504_Gret1	D708
	21	Muscat of Alexandria	D504_Gret1	D709
	22	Muscat Bailey A	D504_Gret1	D710
	23	Ruby Roman	D504_Gret1	D711
	24	Rosario Bianco	D504_Gret1	D712
Tvv1	1	Aki Queen	D505_Tvv1	D701
	2	Campbell Early	D505_Tvv1	D702
	3	Kyoho	D505_Tvv1	D703
	4	Queen Nina	D505_Tvv1	D704
	5	Grosz Krone	D505_Tvv1	D705
	6	Koshu	D505_Tvv1	D706
	7	Concord	D505_Tvv1	D707
	8	Sunverde	D505_Tvv1	D708
	9	Shine Muscat	D505_Tvv1	D709
	10	Suiho	D505_Tvv1	D710
	11	Steuben	D505_Tvv1	D711
	12	Sekirei	D505_Tvv1	D712
	13	Takao	D506_Tvv1	D701
	14	Delaware	D506_Tvv1	D702
	15	Niagara	D506_Tvv1	D703
	16	Nagano Purple	D506_Tvv1	D704
	17	Pione	D506_Tvv1	D705
	18	Fujiminori	D506_Tvv1	D706
	19	Black Beet	D506_Tvv1	D707
	20	Portland	D506_Tvv1	D708

21	Muscat of Alexandria	D506_Tvv1	D709
22	Muscat Bailey A	D506_Tvv1	D710
23	Ruby Roman	D506_Tvv1	D711
24	Rosario Bianco	D506_Tvv1	D712

Supplemental Table 3. Primers used for PCR amplification

Primer name	Sequence (5′→3′)
VINE1-PBS	CAAGATAACTATGGCTCTGATACCA
Cl155	GCTCATGCATGGGAACTTCTTC
Cl160	CAGCTATACGAGCTGATGGAAC
